# 2328. COVID-19 and CLABSI: Riding the Pandemic Waves with an Active Infection Prevention Intervention

**DOI:** 10.1093/ofid/ofad500.1950

**Published:** 2023-11-27

**Authors:** Rama Thyagarajan, Kristin E Mondy, Lisa K Sturm, Coutney Watson, Karl Saake, Collin Miller, Frederick Masoudi, Mohamad G Fakih

**Affiliations:** Dell Seton Medical Center, Department of Internal Medicine, Dell Medical School, University of Texas at Austin, Leander, Texas; University of Texas Dell Medical School, Austin, Texas; Ascension, Farmington Hills, MI; Ascension Data Science Institute, Pittsburgh, Pennsylvania; Ascension, Farmington Hills, MI; Ascension Health, St. Louis, Missouri; Ascension/ Chief Science Officer, VP of Research and Anlaytics, St. Louis, Missouri; Ascension, Farmington Hills, MI

## Abstract

**Background:**

The coronavirus disease 2019 (COVID-19) pandemic has had a considerable impact on central line-associated bloodstream infections (CLABSI) in US hospitals.

**Methods:**

We evaluated the impact of COVID-19 on CLABSI in 86 hospitals of a single system with an active infection prevention program. We compared pandemic CLABSI events and associated organisms, using rates and standardized infection ratio (SIR) accounting for changes based on COVID-19 prevalence, to pre-pandemic levels. The pandemic period was further divided into four waves based on the dominant COVID-19 strain. Late in the Delta wave, a system wide re-focus on a CLABSI bundle was implemented, with reformation of multi-disciplinary teams, education and monitoring, direct feedback to caregivers, and increased visibility of CLABSI at all levels.

**Results:**

There were 1,895,534 central line-days with 1,343 CLABSI events in the entire study period. CLABSI rates increased from 0.59 pre-pandemic to 0.76 per 1,000 line-days during the pandemic (28.8% relative increase (p<0.001). Cumulative coagulase-negative staphylococcus (CNS) CLABSI increased by 102% from 0.09 to 0.19 events per 1,000 line-days (p< 0.001), and *Candida sp.* by 39.9% from 0.15 to 0.22 per 1,000 line-days (p=0.006) (Figure 1). Hospitals with monthly COVID-19 prevalence ≥ 20% had a SIR CLABSI that was 2.20 times higher compared to those with < 5% COVID-19 prevalence during pandemic waves (p< 0.001). With the intervention, CLABSI rates subsequently dropped, approximating pre-pandemic levels (Figure 2).

**Figure 1:**
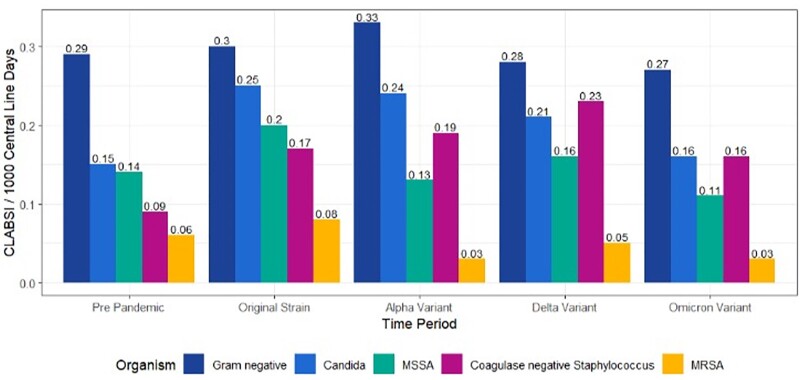
Comparison of pathogens associated with CLABSI across pre-pandemic and four COVID-19 pandemic waves

**Figure 2:**
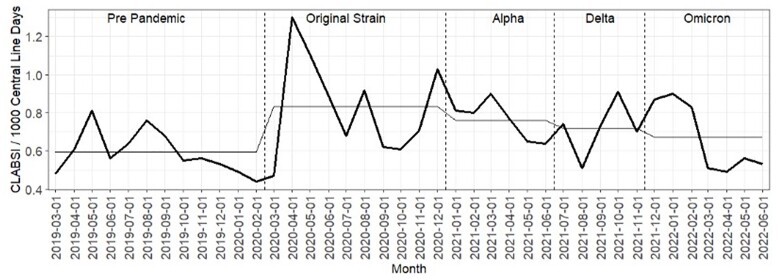
CLABSI rates/1000 central line days across the pre-pandemic and four COVID-19 pandemic waves

**Conclusions:** A high hospital prevalence of COVID-19 was associated with increased CLABSI events in early pandemic waves. Improvements in CLABSI rates were observed in later waves concurrently with re-focused prevention efforts, underscoring that enhanced attention to infection prevention processes can achieve pre-pandemic levels of CLABSI events.

**Disclosures:**

**All Authors**: No reported disclosures

